# Group antenatal care (Pregnancy Circles) for diverse and disadvantaged women: study protocol for a randomised controlled trial with integral process and economic evaluations

**DOI:** 10.1186/s12913-020-05751-z

**Published:** 2020-10-07

**Authors:** Meg Wiggins, Mary Sawtell, Octavia Wiseman, Christine McCourt, Sandra Eldridge, Rachael Hunter, Ekaterina Bordea, Connor Mustard, Ainul Hanafiah, Bethan Hatherall, Vivian Holmes, Anita Mehay, Helliner Robinson, Cathryn Salisbury, Lorna Sweeney, Kade Mondeh, Angela Harden

**Affiliations:** 1 Social Research Institute, University College London, 27 Woburn Square, London, WC1H 0AA UK; 2 School of Health Sciences, City, University of London, London, UK; 3 Queen Mary University of London, Pragmatic Clinical Trials Unit, London, UK; 4 Research Department of Primary Care and Population Health, University College London, London, UK; 5 University College London, Institute of Clinical Trials & Methodology, London, UK; 6 Institute for Health and Human Development, University of East London, London, UK; 7 Barts Health NHS Trust, London, UK

**Keywords:** Inequalities, Prenatal, Pregnancy, Midwives, Group care, Statistics and research methods

## Abstract

**Background:**

Group antenatal care has been successfully implemented around the world with suggestions of improved outcomes, including for disadvantaged groups, but it has not been formally tested in the UK in the context of the NHS. To address this the REACH Pregnancy Circles intervention was developed and a randomised controlled trial (RCT), based on a pilot study, is in progress.

**Methods:**

The RCT is a pragmatic, two-arm, individually randomised, parallel group RCT designed to test clinical and cost-effectiveness of REACH Pregnancy Circles compared with standard care. Recruitment will be through NHS services. The sample size is 1732 (866 randomised to the intervention and 866 to standard care). The primary outcome measure is a ‘healthy baby’ composite measured at 1 month postnatal using routine maternity data. Secondary outcome measures will be assessed using participant questionnaires completed at recruitment (baseline), 35 weeks gestation (follow-up 1) and 3 months postnatal (follow-up 2). An integrated process evaluation, to include exploration of fidelity, will be conducted using mixed methods. Analyses will be on an intention to treat as allocated basis. The primary analysis will compare the number of babies born “healthy” in the control and intervention arms and provide an odds ratio. A cost-effectiveness analysis will compare the incremental cost per Quality Adjusted Life Years and per additional ‘healthy and positive birth’ of the intervention with standard care. Qualitative data will be analysed thematically.

**Discussion:**

This multi-site randomised trial in England is planned to be the largest trial of group antenatal care in the world to date; as well as the first rigorous test within the NHS of this maternity service change. It has a recruitment focus on ethnically, culturally and linguistically diverse and disadvantaged participants, including non-English speakers.

**Trial registration:**

Trial registration; ISRCTN, ISRCTN91977441. Registered 11 February 2019 - retrospectively registered. The current protocol is Version 4; 28/01/2020.

## Background

Antenatal care is an important public health priority which can promote good health both in pregnancy and during the life-course of women and their children. However, limitations to traditional models of care have been documented [1]. Those from ethnic minority and socially disadvantaged populations are known to experience both poorer access to, and quality of, antenatal care [2]. Adverse pregnancy outcomes are linked with such inequity [3].

Traditional antenatal care, delivered by midwives, is on a one-to-one basis, but antenatal care delivered by midwives on a group basis, has been implemented in several countries with some success. Examples include in the UK [4], Australia [5], the US [6], Iran [7] and elsewhere. Evidence on group care shows increased women’s satisfaction [7, 8] as well as potential better health and safety outcomes [6, 8, 9]. Furthermore, delivery of group antenatal care to vulnerable groups [10] has been shown to be successful, with evidence of possible improved outcomes for specific groups. Qualitative studies have also indicated potential added advantages arising from having a mixed group of participants [4].

### What does group antenatal care involve?

In this model of antenatal care, health professionals (usually, but not always, midwives) facilitate groups and provide continuity of care to around 8–12 pregnant women with similar estimated due dates. Group sessions replace traditional one-to-one appointments and last about two hours. Conventional facets of antenatal assessment such as clinical checks are combined with information sharing and peer support through group discussion led by participants. Group care was developed to address some of the negative experiences of antenatal care reported by women. These include problems that are particularly challenging, in standard care, for more vulnerable populations such as the very limited amount of time that pregnant women spend with care givers and lack of continuity of carer [11–13]. The opportunity group care offers to build social support is likely to be particularly beneficial for those whose existing support networks are limited or non-optimal [14–16].

### Why might group antenatal care be beneficial?

It is hypothesised that this group approach will promote women’s empowerment, enabling them to take a more active role in their care, supporting informed decision making and facilitating more effective tailoring of their antenatal care to their own requirements. The group approach encourages women to engage in self-monitoring (i.e. checking their own urine and blood pressure) with the aim of increasing their knowledge and confidence [4]. In pregnancy, an increased sense of autonomy and wider choice have been linked to a sense of greater control around birthing, which in turn can increase women’s satisfaction with giving birth. Better birth experiences have the potential to impact on the wellbeing of women and their children [17–20]. Additionally, recent evidence has shown that midwives trained to deliver group antenatal care report satisfaction with working in this way [21].

Outcomes of group antenatal care have not been rigorously tested in the context of universal health care, nor specifically in the UK within the context of the NHS. Systematic reviews of group antenatal care conclude that there is a need for additional rigourous studies to determine whether previous positive findings are applicable in different contexts [8, 10, 22]. The study described here will aim to assess the clinical effectiveness of the REACH Pregnancy Circles model of group antenatal care in a randomised controlled trial (RCT) with integrated economic and process evaluations. The trial will run in areas of the UK that are diverse (in terms of ethnicity, culture and language) and/or economically disadvantaged. REACH Pregnancy Circles is a bespoke model of group care which is designed to be responsive to individual service needs and directly translates many of the recommendations of the current UK national policy for maternity services [1].

This study is part of a NIHR-funded Programme Grant for Applied Research, the REACH Pregnancy Programme (Reference RP-DG-1108-10,049), which aims to improve women’s access to, engagement with, and experience of antenatal care. The Programme comprises four main components including this trial and a two-stage feasibility/pilot study preceding it [4, 21, 23]. This preparatory work, in line with best practice recommendations [24], has enabled the research team to understand and address the local and UK national challenges that any group-based model of antenatal care needs to be tailored to meet, as well as to develop and test the methods for the full trial.

## Methods

### Aims

The REACH Pregnancy Circles trial aims to assess the following in ethnically, culturally and linguistically diverse and disadvantaged areas of the UK:

- Whether Pregnancy Circles (group-based antenatal care) improves the health of babies compared with the standard individual model of antenatal care
- Whether attending Pregnancy Circles improves maternal outcomes such as empowerment and post-natal psychological wellbeing, as well as increasing women’s satisfaction with antenatal care
- Cost-effectiveness, intervention mechanisms and acceptability of Pregnancy Circles care to women and staff and issues relevant to future sustainability
  and wider implementation in the NHS.

### Trial design

The REACH trial is a pragmatic, two-arm, individually randomised, parallel group RCT. It involves an intention-to-treat (ITT) comparison of the Pregnancy Circles group antenatal care intervention with standard care.

The trial includes an integral process and economic evaluation. The process evaluation is a mixed method study, aligned with Medical Research Council guidance on process evaluations of complex interventions [25]. It will include in depth exploration in three case study sites involving questionnaires and semi-structured interviews with service users, midwives and other stakeholders and observations of antenatal care; as well as utilising cross site monitoring data, reflective diaries and minutes from meetings to explore facilitators and barriers to implementation, measure uptake, retention and fidelity to the intervention.

The health economics evaluation will use a cost-effectiveness analysis of the incremental cost per Quality Adjusted Life Year (QALY) of the Pregnancy Circles intervention compared with standard care. It will also calculate the incremental cost per additional ‘healthy and positive birth’ for group antenatal care compared to control, using an innovative composite measure.

An internal pilot to check the feasibility of rates of recruitment at scale will be conducted within the recruitment period. Data from the internal pilot will be presented to the Trial Steering Committee to support decisions on trial processes and continuation.

### Setting

The trial will be carried out within the maternity services of around 12 NHS trusts in London and other areas of England. A number of ‘Pregnancy Circles’ (i.e. one group of women who have their antenatal appointments together) will be run within the catchment areas of each of these trusts by midwives from the local service. It is anticipated that around 7–14 Pregnancy Circles will be run by each service.

### Participants

**Fig. 1:**
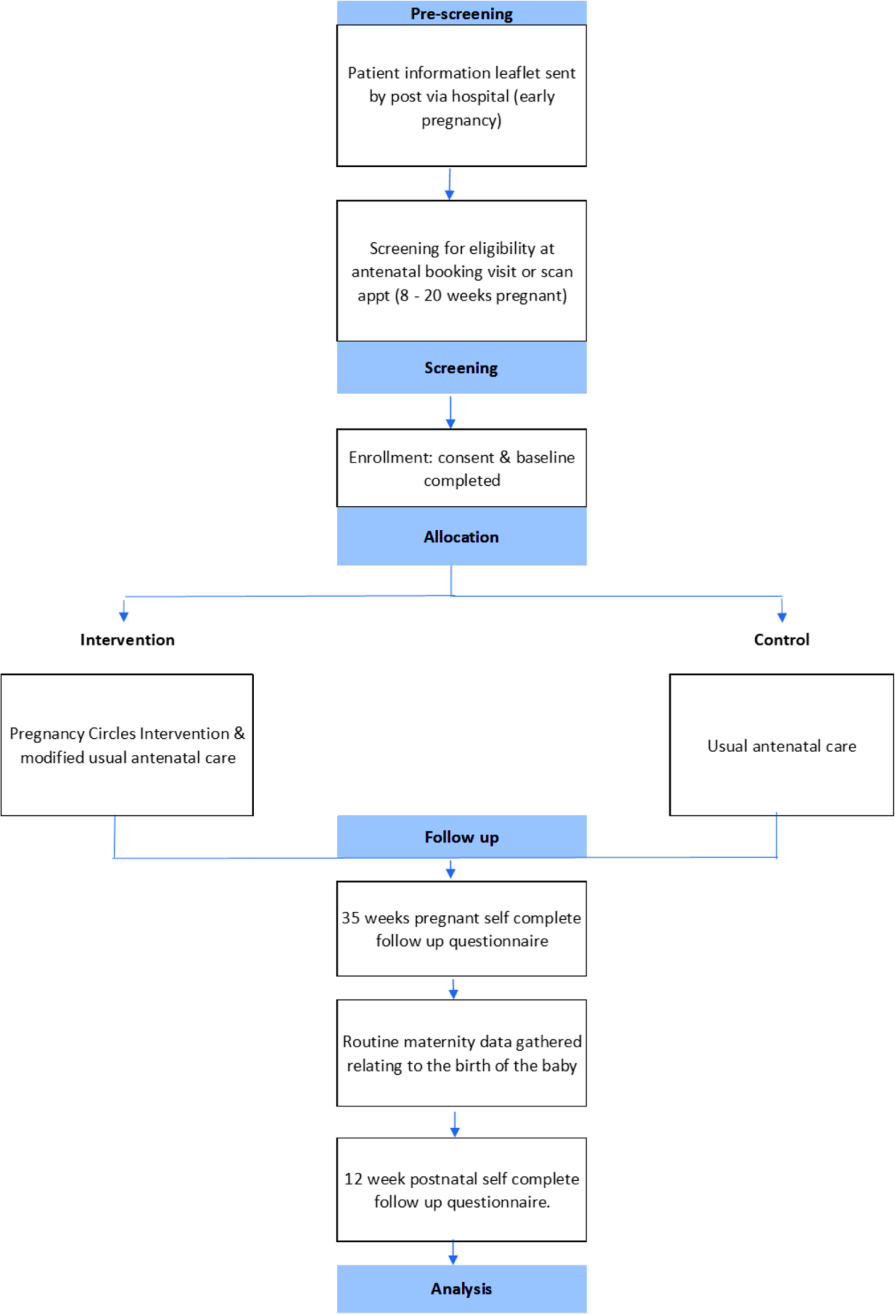
Participant Flow

Women who are currently pregnant and registering for antenatal care, with any of the included maternity services before 20 weeks of pregnancy (in order to capture late bookers who are likely to be more vulnerable), will be approached to take part in the trial. Fig. 1 shows participant flow through the trial.

### Eligibility criteria

Pregnant women will be eligible for inclusion in the study if they:

- are 16 years old and over
- are part of the cohort of women cared for by the team delivering the intervention (generally one geographical area)
- have an estimated delivery date that fits with those of a proposed group
- do not have a documented learning disability.

The trial is designed to be as inclusive as possible and will include women who: are primiparous and multiparous; “low” and “high” obstetric risk (e.g. diabetic); have obstetric complications (e.g. multiple pregnancies); have additional physical or social needs; do not speak English.

As a rule, women will be offered participation so that they can make their own decision about this. Where specialist pathways are in place (e.g. diabetes, twins, teenagers etc.) referral to these services will be made. Women can choose to attend Pregnancy Circles in addition to specialist services. Local clinicians will make decisions about offering participation to the most vulnerable women on a case by case basis (for example where there are child protection concerns), depending on local pathways for vulnerable women.

We learnt from the pilot study that managing the number of languages spoken in a Pregnancy Circle is important. Therefore, before recruitment to each Pregnancy Circle starts, facilitating midwives at each site will limit the number of different languages spoken within a circle where interpreter support is required. Once recruiters have reached this ‘cap’ for each circle, any subsequent pregnant woman who meets the inclusion criteria but requires interpreter support for a language different from those already included in a circle, will be deemed ineligible.

Midwife facilitators will be eligible to take part if they are employed by the maternity services involved in the trial and have attended the study specific 1-day training provided by the research team, or been trained by an experienced facilitating midwife.

### Randomisation, blinding and bias

Randomisation will be carried out by recruiters in the clinic, immediately following consent and baseline questionnaire completion, using a centralised online randomisation system developed by the Pragmatic Clinical Trials Unit (PCTU) at Queen Mary, University of London. Participants will be told their allocation status straight away. Randomisation will be stratified by trial site and how well a woman speaks English and participants will be randomised in equal numbers to intervention and control arms. Women in the control arm will proceed with standard care as per local procedures. Those in the intervention arm will receive a welcome pack detailing information on the venue and dates/times and an outline of the content of all their Pregnancy Circle sessions as well as contact details for their named facilitating midwives.

The Trial Steering Committee (TSC), study statisticians, health economists and chief investigator will be blinded to treatment allocation during the trial. Some members of the central research team, all members of site teams and participants cannot not be blinded due to the nature of the randomisation procedure and the intervention.

There is a possible risk of facilitator bias as midwives delivering the intervention may change their practice in a standard care context as well as in the Pregnancy Circles, for example in terms of being more ‘woman-led’. The chance of such contamination will be limited by the fact that several key elements of the Pregnancy Circles model cannot be reproduced in a standard care setting (e.g. the social element, the self-checking, the extended time of appointments, the degree of continuity of carer). However, there remains a risk, the level and effects of which will be assessed through the process evaluation.

### Site selection and participant recruitment

The key issues, in terms of selection of potential trial sites, is that they deliver maternity services in areas with high levels of poverty and/or ethnic and language diversity. Sites that meet this criterion will be asked to ensure that, on average, they have sufficient numbers of antenatal bookings to support the recruitment target, based on our learning from our pilot trial. This target is 24 recruited participants per month randomised 1:1 across trial arms. Aiming for an allocation of 12 to the intervention arm allows for the possibility of attrition (e.g. due to miscarriage) while still maintaining an optimum number of 8–10 women per Pregnancy Circle.

Participant recruitment in the sites will be carried out by staff funded by local Clinical Research Networks (CRN) and will be conducted at either the antenatal booking appointment and/or first dating-scan appointment. The decision on which appointment to target will be based on how maternity services are arranged in the trust with the aim of maximising the numbers of eligible women research staff can access. These appointments usually take place between 8 and 13 weeks of pregnancy although sites will be encouraged to approach late bookers up to 20 weeks pregnant.

Clinic lists will be scrutinised in advance of a clinic and women on the list who appear to fit the inclusion criteria will be identified. The patient information sheet will be provided to these potential participants, either in the post before they attend their booking or scan appointment, or while they are waiting in the clinic for their appointment.

At the booking or scan appointment, the recruiter will assess eligibility, take written informed consent and request completion of the baseline questionnaire. This will be a paper document replacing a planned online version provided via the electronic patient recorded outcome tool (REDCap). The online version was found to be impractical when piloted in the clinic setting at the start of recruitment. Randomisation will then be carried out. Women who are unsure about participation will be invited to take study documents away and have a few days for consideration. Completion of documents and randomisation can then be carried out, via liaison with recruiters, face to face or by remote methods adhering to a procedure that ensures best consent practice and GDPR compliance. A £10 voucher will be provided to each recruit as recognition of their time and effort.

Language support for recruitment will be provided for women who meet the eligibility criteria who do not speak English. Options for language support at each site include some or all of the following: interpreters employed by participating NHS trusts; researchers who speak required languages; a phone interpreting service; or informal support from family/other pregnant women. Wherever possible women will be offered their preferred route.

In advance of the study starting, a number of meetings will be run by the research team for service side staff to provide information and discuss support needed.

### Intervention

The Pregnancy Circle model will consist of eight antenatal group sessions, reflecting the standard schedule for antenatal care in the UK, each of which will last two hours. Women will have the usual booking appointment, one-to-one, and then the first Pregnancy Circle session will take place at approximately 16 weeks of pregnancy. Subsequent sessions will adhere to the standard NHS antenatal care schedule for primigravida women [26]. In addition, participants will be invited to a postnatal reunion group held approximately 1 month after the birth. Midwives will follow the usual NICE guidelines in terms of referrals, safeguarding and the quality of maternity care provided.

Two midwives (or in some cases a midwife and a maternity assistant) will facilitate each Pregnancy Circle group session. Face-to-face interpreters will attend each session where required. Facilitating midwives will receive a bespoke one-day training course and ongoing support, from research midwives in the central research team, to facilitate the Pregnancy Circles. A manual will be provided as a source of guidance for facilitators. Continuity of carer will be aimed for, with the same two facilitators planned for all the sessions for a Pregnancy Circle, with one trained alternate in case of illness or annual leave, and their Pregnancy Circles time will be included on the service roster.

**Fig. 2:**
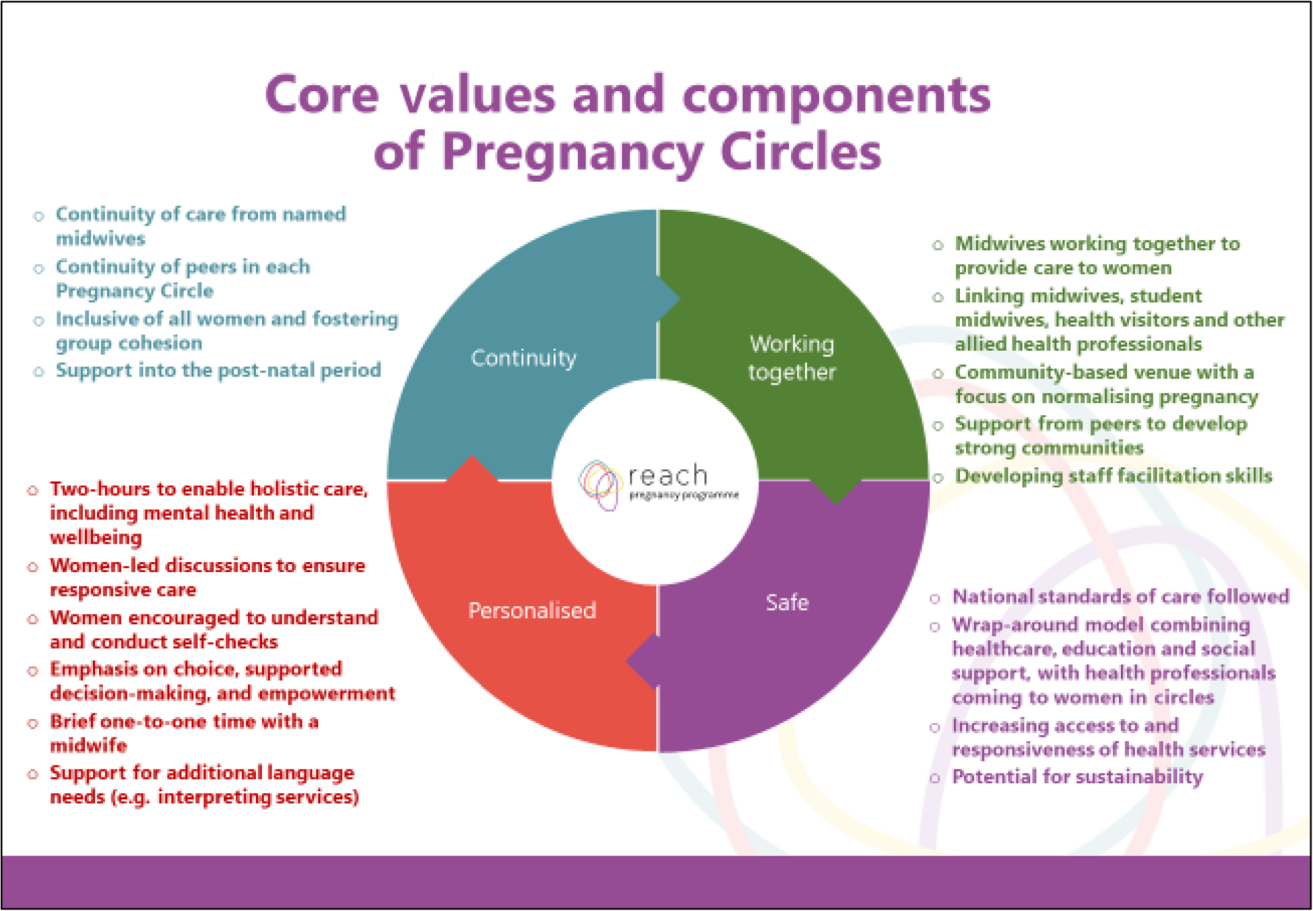
Core Values of the Pregnancy Circles intervention

Each Pregnancy Circle session will begin with ‘self-care monitoring activities’ (e.g. women testing their own blood pressure and urine). Short (3–5 min) one-to-one individual health checks will be carried out in the group space with one of the midwives while the second midwife will facilitate the rest of the group engage in a group discussion. The content of group discussions will follow the values outlined in Fig. 2. They will be woman-led, with the facilitating midwives suggesting any essential topics that need to be covered (in line with national and local clinical guidelines). Women can request more privacy for one-to-one time, as required. How and when the women’s partners participate in the sessions will be decided by the women themselves during the first circle.

A woman who wants to stop receiving group during pregnancy will change to standard care. Facilitating midwives will follow-up non-attenders to ascertain their reasons for this, following the usual local protocol.

### Patient and public involvement

Patient and Public involvement (PPI) to identify priorities and preferences around antenatal care, the proposed intervention and trial processes, was central to the feasibility work that has shaped the trial. The full trial mechanisms have been designed to ensure close working with PPI co-investigators and representatives, who are members of the trial management structures, on all issues related to trial conduct. Furthermore, PPI co-investigators will be involved in aspects of the qualitative data analysis. The research team’s close contact with local Maternity Voices Partnerships will be utilised to consult more widely on specific issues as the trial progresses. PPI co-investigators and practitioners from trial sites will be involved in creating and delivering the study dissemination plans. The central research team will comprise both academics and health care practitioners.

### Outcome measures

Potential outcomes and appropriate methods for utilising them were piloted [23].

The primary outcome is a ‘healthy baby’ composite measured at 1 month postnatal using routine maternity data. The composite is comprised of the following four components:

1. Live baby *(no stillbirth after 24 completed weeks of pregnancy and no neonatal death within 28 days of the birth)*
2. Born at term *(37 weeks and above)*
3. Appropriate weight for gestational age *(GROW centile > 9.99 & < 90.01)* [27]
4. Not admitted for neonatal intensive care

A baby is considered a ‘healthy baby’ if the answer to all 4 components is ‘yes’.

The secondary outcomes are listed in Table 1, which specifies the time points when collected and the standard measures being utilised, where appropriate.

**Table 1:** Secondary outcomes for Pregnancy Circles Trial

Domain	Validated measure (if using)	Baseline	First follow up - 35 weeks gestation	Birth – routine maternity data	Second follow up - 3 months postnatal
Women’s empowerment	Pregnancy-related Empowerment Scale (PRES) [28]	✗	✓	✗	✗
Women’s satisfaction with maternity care	Friends and Family Test [29]	✗	✓	✗	✓
Continuity of antenatal care		✓	✓	✗	✗
Attendance at antenatal care		✓	✓	✓	✗
Health service usage from patient records		✗	✗	✓	✗
Health service usage (self-reported)		✗	✓	✗	✓
Social support	The Duke-UNC Functional Social Support Questionnaire [30]	✓	✓	✗	✓
Self-efficacy	Pearlin Mastery Scale [31]	✓	✓	✗	✓
Prenatal stress	Revised Prenatal Distress scale [32]	✓	✓	✗	✗
Spontaneous vaginal delivery (SVD)		✗	✗	✓	✓
Caesarean delivery		✗	✗	✓	✓
Infant birth weight, defined as low if less than 2500 g		✗	✗	✓	✗
Place of birth		✗	✗	✓	✗
Breast feeding initiation		✗	✗	✗	✓
Breast feeding continuation and exclusivity		✗	✗	✗	✓
Postnatal depression	Edinburgh Postnatal Depression Scale (EPDS) [33]	✗	✗	✗	✓
Health Literacy	Health Literacy Questionnaire (HLQ) [34] (1 domain)	✗	✓	✗	✗
Postnatal symptoms	Postnatal symptoms checklist (National Maternity Survey 2010) [35]	✗	✗	✗	✓
Emotional wellbeing	Short Warwick-Edinburgh Mental Wellbeing Scale (SWEMWBS) [36]	✓	✓	✗	✗
Live baby (i.e. no stillbirth after 24 completed weeks of pregnancy and no neonatal death within 28 days of the birth)		✗	✗	✓	✗
Born at term (37 weeks and above)		✗	✗	✓	✗
Appropriate weight for gestational age (GROW centile > 9.99 & < 90.01)		✗	✗	✓	✗
Not admitted for neonatal intensive care		✗	✗	✓	✗
Health related quality of life	EQ5D-5 L [37]	✓	✓	✗	✓

The pilot trial showed that the completeness of the routine data and the burden of questionnaires were acceptable.

### Data collection and management

Data management for the trial will be overseen by the PCTU. Information related to participants will be kept confidential and managed in accordance with the Data Protection Act, NHS Caldecott Principles, The Research Governance Framework for Health and Social Care, and the conditions of research ethics committee approval. Participant survey data will be captured on a REDCap database which will be developed, supported and securely hosted by the PCTU. REDCap is a secure web-based tool that requires researchers to use a two-step verification process to gain access.

The self-complete baseline questionnaire data collected at recruitment will be entered onto REDCap by the central research team following receipt of paper baseline questionnaires sent securely from trial sites. Questionnaires are identified by participant ID number only and will be transported and stored separately from any paperwork with identifiable information on, including consent forms. All participant paperwork will be stored in locked filing cabinets within locked rooms.

Participants will complete follow-up outcomes questionnaires at 35 weeks pregnant and 3 months postnatal. Participants who provided an email address will be sent a survey link via REDCap; paper versions with reply envelopes will be sent to participants without email addresses. Where a woman requires language support, a researcher will work with an interpreter to arrange completion over the phone or face-to-face in a setting of her choice. In advance of contacting women about these questionnaires, the research team will check with site staff that there are no reasons why a woman should not be approached. Non responders will be reminded once electronically and then followed up on the telephone to encourage completion. All women will be given a £10 voucher for each of the two outcomes questionnaires they complete.

To access routine maternity data, researchers will work with local sites for the transfer of electronic patient record data, supplemented where there are gaps via access to paper maternity notes. The research team will supply the trust informatics team with a list of participant hospital numbers and study ID numbers, and a proforma for required data. All anonymised electronic routine patient data will be electronically transferred from the participating trusts directly into the PCTU’s safehaven as per the PCTU’s dedicated secure file transfer protocol. Where there are gaps in the primary outcome electronic data, local maternity staff who are blinded to the allocation to study group will conduct an audit by extracting data manually from the participants’ hospital paper records within the hospital setting. An endpoint committee will be set up to assess any cases where there are uncertainties around primary outcome status. Members of the research team and a minimum of two clinicians will be on this committee.

Intervention attendance will also be collected using records kept by facilitating midwives. Participants can withdraw from either just the intervention or from the whole study, this decision will be recorded. If the participant just withdraws from the intervention, they will be followed-up. If they fully withdraw from the study, no further data will be collected.

Audio recordings and transcripts of the research interviews will be stored on the secure servers at City, University of London or University of East London (UEL). Confidentiality of personal data will be ensured through the use of anonymisation and pseudonymisation techniques.

### Sample size

Originally at the onset of the trial, the primary outcome was planned to be spontaneous vaginal birth (SVB). An initial calculation for SVB as the primary outcome produced a sample size of 2120 women, assuming a difference of proportion of 70.2 to 77.5%, with 25% drop-out, mean group size of 10 women, intra-cluster correlation coefficient (ICC) of 0.1 and 80% power. However, the ethics committee requested a change in primary outcome as they had concerns that using SVB might influence midwives and women in mode of delivery decisions to the detriment of health outcomes. As such, the ‘healthy baby’ composite was adopted instead.

Previous evidence relating to the variables in our ‘healthy baby’ composite suggests expected decreases in rates of small for gestational age by between 20 and 30% [38, 39], pre-term birth by 29–54% [6, 38, 40], and neonatal intensive care admissions by up to 65% [9]. We therefore conservatively estimate that the rate of unhealthy babies will decrease by 25%. Analyses of routine data from our pilot study NHS trust indicates that 31% of babies would experience these adverse outcomes. A decrease of 25% equates to an 8%-point decrease to 23%. Correspondingly, this translates directly as an increase in overall rates of healthy babies by 8% points from 69 to 77%. Our Programme Steering Committee (PSC) and stakeholders have indicated that the healthy baby composite and anticipated effect size reflects a clinically relevant and potentially practice-changing outcome.

For the primary outcome of the healthy baby composite, to detect an increase in the proportion of babies born “healthy” by 8% between the control and intervention arm, with 90% power and a 5% significance level would require at least 866 women per arm (1732 total). This assumes an outcome proportion of 69% in the control arm and 77% in the intervention arm. Given that the intervention is for group antenatal care, this sample size calculation also accounts for clustering within the intervention arm, with an ICC of 0.05, using mean group sizes of 8 with cluster size variability assuming a Poisson distribution for cluster size. This calculation was informed by the findings of the pilot trial and given that the primary outcome is from electronic routine maternity data, it therefore assumes 10% drop-out in both arms. When applying the updated sample size (1732 total) to SVB and the same difference of proportion a power of 84.7% was calculated when using the updated parameters (ICC of 0.05, mean group size of 8 and 10% drop out).

### Data analysis

A statistical analysis plan (SAP) will be written and signed off before any allocation codes are provided to the statistician analysing the trial and all those involved in developing the analysis plan will remain blind to allocation codes. Furthermore, the mechanism for dealing with missing data, any sub-group analyses and any sensitivity analyses will be outlined in the SAP. The analysis plan will be reviewed by the independent statistician on the TSC. The randomisation stratification factors will be used as covariates in the models for the between treatment analysis. If models are to be adjusted for other covariates, then these will be clearly stated in the statistical analysis plan. All statistical tests will be two-tailed with alpha = 0.05 and analyses will be performed on an ITT basis. Baseline data, demographic and wellbeing information will be described and summarised overall and by treatment group.

The primary outcome data for the ‘healthy baby’ composite will be extracted via a postpartum maternity records audit and be analysed using a logistic random effects model with a random intercept estimating a cluster specific effect in both arms. In the intervention arm, within Pregnancy Circle correlation will be accounted for and in the control arm each participant will be modelled as a cluster of size 1. An odds ratio and associated 95% confidence interval will be presented. Secondary outcomes will be analysed using the same mixed effects model accounting for Pregnancy Circle correlation in the intervention arm and will be presented with appropriate treatment effect estimates (odds ratios, mean differences) and associated 95% confidence intervals, based on the outcomes themselves (i.e. binary/continuous scales).

All analyses will be performed based on available participant data for the primary and secondary outcomes of interest. There are no planned interim statistical analyses, with no highlighted stopping rules in relation to efficacy in this trial.

### Health economics evaluation

The economic evaluation will determine the cost-effectiveness of Pregnancy Circles compared to standard care for the period from conception until three months postpartum, from a health and social cost perspective. The cost of the intervention will include the cost of training and the cost of the Pregnancy Circles. The latter will be calculated based on forms completed by midwives on the duration of the Pregnancy Circles, the number of women in each circle and any additional time required in the set up and running of the circle. Inpatient care in the antenatal and postnatal periods will be collected from patient records in both trial arms. Additional maternity and infant related resource use will be provided via participant completed questionnaires at 35 weeks and three months postpartum as well as via routine maternity data. Resource use will be costed based on published sources and will be presented as descriptive statistics for resources. Differences in costs between the two groups will be calculated using linear regression, adjusting for randomisation stratification factors as covariates and a random intercept for Pregnancy Circle. Ninety five percent confidence intervals will be calculated from bootstrapped results with bias correction.

The NICE recommendation that cost-effectiveness is calculated as the cost per QALY gained [41], is challenging in this study’s context. For the mother, the measurement of QALYs is possible; but from the perspective of the infant, the timing and method of measuring QALYs is contentious and presents methodological difficulties. The EQ-5D-5L has been included at baseline, first and second follow up for the calculation of QALYs in the mother, which will be calculated as the area under the curve adjusting for baseline and randomisation stratification factors with random intercept for Pregnancy Circle. Ninety five percent  confidence intervals will be calculated from bootstrapped results with bias correction. In the pilot trial, the economic evaluation used participant questionnaires to explore additional utility measures. Building on this in this trial, women will be involved in defining what a ‘positive and healthy pregnancy and birth’ is, to enable a calculation of the incremental cost per additional ‘healthy birth’ for Pregnancy Circles compared to standard care, using a new composite measure. The follow up data collected from the pilot trial will be utilised in an assessment of the validity of this new composite measure. In further development work, which is on-going within this trial, algorithms will be developed for how births might be classified on a ‘healthy’ to ‘unhealthy’ continuum. The mean incremental cost per ‘healthy birth’ and the mean incremental cost per QALY will be reported. Cost-effectiveness acceptability curves and cost-effectiveness planes will be constructed based on bootstrapped data as described above. The economic evaluation will handle missing and censored data in line with the methods utilised in the statistical analysis.

### Process evaluation

The aim of the integral process evaluation is to help understand the presence or absence of treatment effects and to identify any unanticipated or unintended effects, including adaptive systems effects. It will also explore women’s experiences of antenatal care as well as potential contamination of the control group. The process evaluation will employ some data collection across all study sites, but in the main will concentrate on three trial sites which will form case studies for more in-depth data collection. These will be purposively selected with the aim of achieving variation of cases.

#### Observations of pregnancy circles and standard care

Non-participant researcher observation will be conducted, utilising a semi-structured observation proforma to record the observations. In each of the case study sites approximately three group sessions (across different Pregnancy Circles) and three standard care consultations will be observed. The aim of observations of the Pregnancy Circles is to capture data to support the measurement of fidelity and for individual consultations to facilitate the reporting of a description of standard care. The Pregnancy Circles to be observed will be purposively selected for diversity of issues including: different languages spoken in the group; partner presence; mix of prima and multi-gravida; stage of pregnancy of the participants; experience of the facilitators in delivering the intervention. In addition, some non-case study sites which warrant specific interest may also be approached for observations. Standard care clinic appointments, conducted by the midwife team delivering Pregnancy Circles, will also be purposively selected to ensure a range of care typical to that area is reflected (e.g. community vs hospital-based). Consent for the observations will be obtained from staff, women and any partners present. If anyone in the group care wishes to withdraw consent their data will be excluded. The observation of standard clinical care will not be carried out where women do not give consent or where interpreting support is needed, but not available.

#### Semi-structured interviews with study participants and key stakeholders

A sample in each case study site of approximately seven Pregnancy Circle and seven control participants will be invited to participate in a one to one semi-structured postnatal interview. The interviews will explore participant’s experiences and satisfaction with their antenatal care and their perceptions of its effects. Sampling will be purposive, to focus on understanding the experiences of clinically and socially high risk and disadvantaged participants. The views and experiences of women who received language support as well as those who have chosen to leave Pregnancy Circles to return to standard care will also be sought. Participants will be interviewed at a time and location of their choice, either over the phone, at home or in a community venue with an appropriate level of privacy. Interviews will last about 30–60 min and will be audio recorded. Women participating in these interviews will be given a £10 voucher as a ‘thank you’ for their time and effort.

In the case study sites, a purposive sample of midwives and key stakeholders (other relevant staff, recruiters, clinical commissioners and patient group representatives) will be offered the opportunity to take part in a brief one to one or paired interview. These will explore perceptions of issues such as delivery of Pregnancy Circles, retention in the groups, sustainability of this model of care, and potential control group contamination. In addition, some midwives and other key stakeholders who are not part of the case studies but who are outliers or practising in a unique way may also be invited to interview.

#### Intervention utilisation, context and background

Monitoring data, including attendance data, will be collected across all trial sites by midwives running the Pregnancy Circles. This will be utilised by the economic evaluation and will also provide information for the process evaluation regarding uptake and retention in the intervention. The process evaluation will also draw on the following documents to provide context and background:

- Closing interviews with link researcher for each study site;
- Field notes from researchers on training sessions run for facilitating midwives;
- Facilitating midwives’ reflective forms;
- Research team processes and implementation records.

#### Process evaluation data analysis

**Fig. 3:**
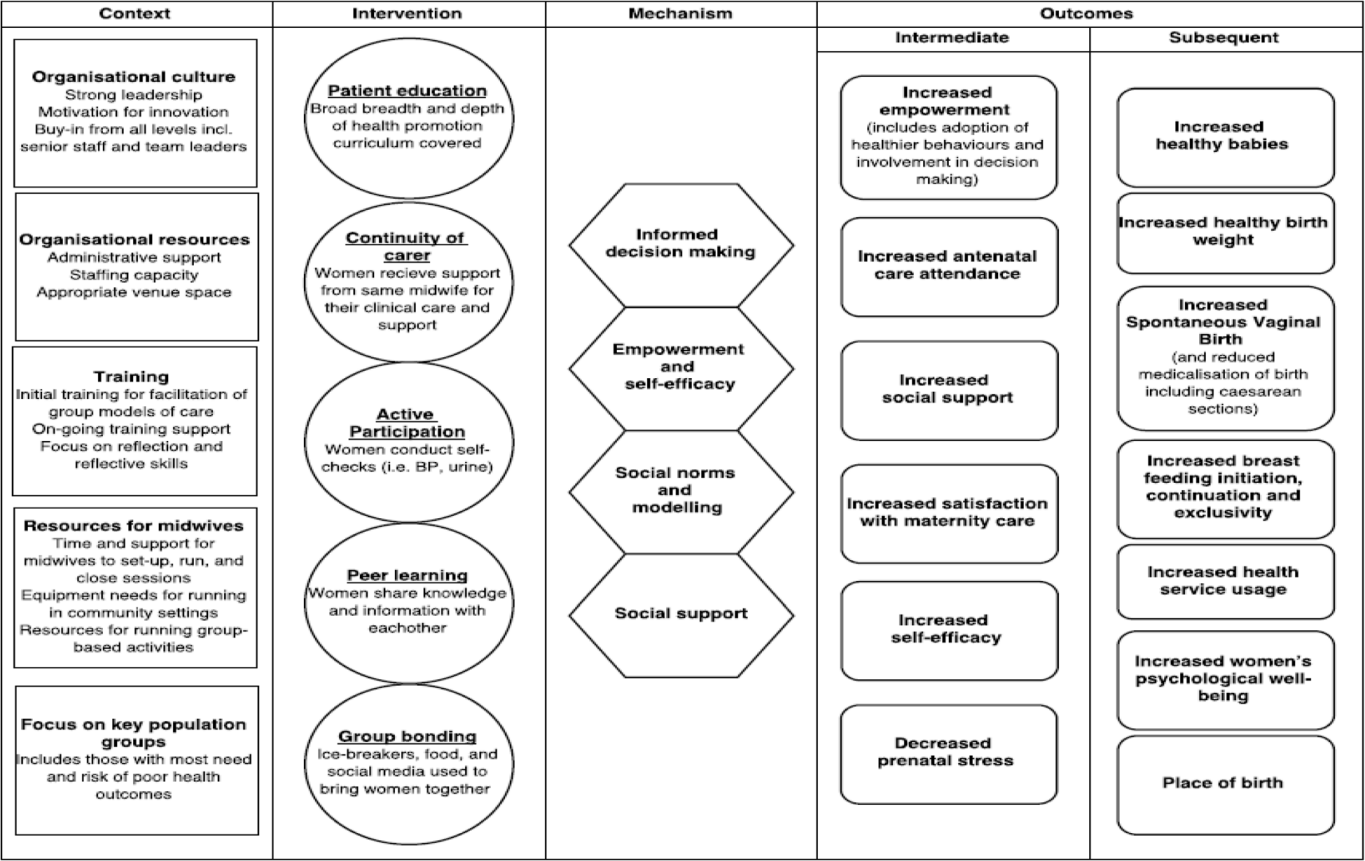
Logic Model

Attendance data will be analysed with SPSS, providing descriptive statistics. All qualitative observational data and interview transcripts will be analysed thematically using NVivo 12, and, with reference to the study logic model (see Fig. 3). The logic model utilises a Context-Mechanism-Intervention framework which draws on realist evaluation principles. Triangulation across data sources will be carried out. The Consolidated Framework for Implementation Research will be used to inform the analysis in relation to implementation of this model of care [42]. In addition, Normalisation Process Theory [43] will inform the analysis of the potential for future integration (‘normalisation’) of this model of care into routine maternity practice within the NHS.

## Governance and safety

### Governance

This trial is one part of a wider NIHR programme grant. The trial is sponsored by the UEL and monitored by both the overall PSC as well as its own TSC. The TSC is responsible for overseeing the trial to ensure; scientific rigour, health service relevance, high ethical and research governance standards. The independent TSC members include a chairperson with maternity trials expertise, a statistician, an economist, a midwifery representative and a PPI representative. The TSC decided a separate data monitoring committee was not needed and approved arrangements for any necessary monitoring and reporting of interim data.

### Safety

This trial is using a serious adverse event (SAE) system. Definitions of SAEs in the study include: death of the participant; life- threatening incident; persistent or significant disability or incapacity; hospitalization for duration of four or more nights; any other safety issue considered medically important, including those affecting the participant’s baby e.g. stillbirth/neonatal death, congenital anomaly or birth defect.

SAEs will be assessed to decide if they are related to the intervention or other trial procedures. If they are the sponsor and research ethics committee will be immediately informed. SAEs are periodically reported to the TSC.

It is standard practice on maternity trials to exclude as SAEs common events in pregnancy that are unlikely to be related to any research procedures. This is particularly relevant in trials of interventions, such as this one, which have a low risk profile.

## Discussion

The REACH Pregnancy Circles trial aims to assess whether group-based antenatal care improves the health of babies compared with the standard individual model of antenatal care in ethnically, culturally and linguistically diverse and disadvantaged areas of the UK.

There are several identified/anticipated key challenges. Firstly, recruiting sufficient participants. The pilot trial that preceded this study demonstrated that the need to recruit approximately 24 women every month, in order to fill each monthly Pregnancy Circle, was likely to require the equivalent of one full-time member of staff recruiting in each trial site for the whole period of recruitment. The study is eligible for NIHR CRN support, including from research midwives for recruitment, but recruitment support is unlikely to be available at the level required by the study. The research team therefore will work creatively with local research departments to ensure the capacity to successfully recruit the required target numbers.

This trial has the added challenge of recruiting less advantaged women as participants, including women who do not speak English. While this is a strength of the trial, and will ensure the intervention reaches the intended population, it also presents practical recruitment challenges. There was significant learning from the pilot trial about how to improve recruitment practices, including recruitment of more vulnerable groups. This learning, which will be incorporated in the full trial included: training recruiters; midwives and administrators not to make assumptions about possible recruits; having local troubleshooting and best practice sessions to ensure best coverage of the appropriate clinics for recruitment; recruiting late bookers; other factors such as easy access to interpreting services and having translations prominently visible on the front of recruitment materials.

While academic-practitioner collaboration in the conduct of RCTs is increasingly common, it remains relatively unusual for allied health professionals, including midwives, to be at the centre of such research. In this study, as with others in the REACH programme [44], the aim will be to have midwives as key collaborators at both central research and site team level (for example as site Principal Investigators). This will not only help to maximise the rigour, relevance and success of the work but also build research capacity in the midwifery profession and NHS trust maternity research teams.

The study started in September 2018 and is scheduled to end in October 2021. At the time of submitting for publication recruitment to the study was on pause due to the restrictions on research in the NHS resulting from the Covid19 pandemic, which may also impact on the study end date.

## Data Availability

At the end of the trial anonymised electronic data, both qualitative and quantitative, that is suitable for open sharing will be stored in the UEL data repository, https://repository.uel.ac.uk, without any restrictions. This data will be reviewed every 5 years in accordance with UEL Research Data Management policy. The data will be kept for the standard retention period of 20 years for clinical trial data. Data that is not suitable for sharing will be securely stored in UEL’s Arkivum data archive. Data is encrypted and only project personnel and UEL Admin staff will have access to it. Any remaining paper-based data that has not been digitised will be kept in a locked filing cabinet in a locked room at UEL or City University for 20 and 10 years respectively (as per institutional requirements).

## Abbreviations

CRN: Clinical Research Nurse
ICC: Intra-cluster correlation coefficient
ITT: Intention to treat
NHS: National Health Service
NIHR: National Institute for Health Research
PCTU: Pragmatic Clinical Trials Unit
PPI: Patient and Public Involvement
QALY: Quality adjusted life year
RCT: Randomised Controlled Trial
REACH: Research for Equitable Antenatal Care and Health
SAE: Serious Adverse Event
SAP: Statistical analysis plan
SVB: Spontanteous vaginal birth
TSC: Trial Steering Committee
UEL: University of East London

## References

[1] National Maternity Review (2016). Better Births: Improving outcomes of maternity services in England.

[2] Lindquist A, Kurinczuk J, Redshaw M, Knight M (2015). Experiences, utilisation and outcomes of maternity care in England among women from different socio-economic groups: findings from the 2010 National Maternity Survey. BJOG.

[3] Knight M, Bunch K, Tuffnell D, Shakespeare J, Kotnis R, Kenyon S, et al. Saving Lives, Improving Mothers’ Care - Lessons learned to inform maternity care from the UK and Ireland Confidential Enquiries into Maternal Deaths and Morbidity 2013–15. Oxford; National Perinatal Epidemiology Unit, University of Oxford; 2019.

[4] Hunter LJ, Da Motta G, McCourt C, Wiseman O, Rayment JL, Haora P (2019). Better together: a qualitative exploration of women’s perceptions and experiences of group antenatal care using focus groups and interviews. Women Birth.

[5] Teate A, Leap N, Schindler Rising S, Homer CSE (2011). Women’s experiences of group antenatal care in Australia - the centering pregnancy pilot study. Midwifery..

[6] Ickovics JR, Kershaw TS, Westdahl C, Magriples U, Massey Z, Reynolds H (2007). Group prenatal care and perinatal outcomes: a randomized controlled trial. Obstet Gynecol.

[7] Jafari F, Eftekhar H, Mohammad K, Fotouhi A (2010). Does group prenatal care affect satisfaction and prenatal care utilization in Iranian pregnant women? 2, 2010, Vol. 39, pp. 52–62. Iran J Public Health.

[8] Catling CJ, Medley N, Foureur M, Ryan C, Leap N, Teate A (2015). Group versus conventional antenatal care for women. Cochrane Database Syst Rev.

[9] Carter EB, Barbier K, Sarabia R, Macones GA, Cahill AG, Tuuli MG (2017). Group versus traditional prenatal care in low-risk women delivering at term: a retrospective cohort study. J Perinatol.

[10] Byerley BM, Haas DM (2017). A systematic overview of the literature regarding group prenatal care for high-risk pregnant women. BMC Pregnancy Childbirth.

[11] McCourt C, Pearce A (2000). Does continuity of carer matter to women from minority ethnic groups?. Midwifery..

[12] Bulman K, McCourt C (2002). Somali refugee women’s experiences of maternity care in West London: a case study. Crit Public Health.

[13] Rayment-Jones H, Murrells T, Sandall J (2015). An investigation of the relationship between the caseload model of midwifery for socially disadvantaged women and childbirth outcomes using routine data – a retrospective, observational study. Midwifery..

[14] Rayment-Jones H, Harris J, Harden A, Khan Z, Sandall J (2019). How do women with social risk factors experience United Kingdom maternity care? A realist synthesis. Birth..

[15] Wedin K, Molin J, Crang Svalenius EL (2010). Group antenatal care: a new pedagogic method for antenatal care - a pilot study. Midwifery..

[16] Andersson E, Christensson K, Hildingson I (2013). Mothers’ satisfaction with group antenatal care versus individual antenatal care -- a clinical trial. Sex Reprod Healthc.

[17] Gottvall K, Waldenstrom U (2002). Does a traumatic birth experience have an impact on future reproduction?. BJOG..

[18] Waldenstrom U (2003). Women’s memory of childbirth at two months and one year after the birth. Birth..

[19] National Institute for Health and Clinical Excellence (NICE). Antenatal and postnatal mental health. Clinical management and service guidance CG192.[online]: The British Psychological Society and The Royal College of Psychiatrists; NICE; 2014 [updated 2020].

[20] All Party Parliamentary Group (APPG) for Conception to Age 2 – The First 1001 Days. Building Great Britons. London: APPG; 2015.

[21] Hunter L, Da Motta G, McCourt C, Wiseman O, Rayment JL, Haora P (2018). It makes sense and it works’: maternity care providers’ perspectives on the feasibility of a group antenatal care model (pregnancy circles). Midwifery..

[22] Ruiz-Mirazo E, Lopez-Yarto M, McDonald SD (2012). Group prenatal care versus individual prenatal care: a systematic review and meta-analyses. J Obstet Gynaecol Can.

[23] Wiggins M, Sawtell M, Wiseman O, McCourt C, Greenberg L, Hunter R (2018). Testing the effectiveness of REACH pregnancy circles group antenatal care: protocol for a randomised controlled pilot trial. Pilot Feasibility Studies.

[24] Medical Research Council. Developing and evaluating complex interventions: new guidance. London: Medical Research Council; 2019.

[25] Moore G, Audrey S, Barker M, Bond L, Bonell C, Hardeman W (2014). Process evaluation of complex interventions: UK Medical Research Council (MRC) guidance.

[26] National Institute for Health and Care Excellence (NICE) (2008). Antenatal care for uncomplicated pregnancies: Clinical guideline [CG62].

[27] Gardosi J, Francis A, Turner S, Williams M (2018). Customized growth charts: rationale, validation and clinical benefits. Am J Obstet Gynecol.

[28] Klima CS, Vonderheid SC, Norr KF, PC G (2015). Development of the Pregnancy-related Empowerment Scale. Nurs Health.

[29] The Friends and Family Test. Publications Gateway Ref. No. 03548. Updated March 2015 (Original Version July 2014). http://www.england.nhs.uk/wpcontent/uploads/2015/07/fft-guidance-160615.pdf (accessed 15 July 2020).

[30] Broadhead WE, Gehlbach SH, DeGruy FV, Kaplan BH (1988). The Duke-UNC functional social support questionnaire. Measurement of social support in family medicine patients. Med Care.

[31] Pearlin LI, Schooler C (1978). The structure of coping. J Health Soc Behav.

[32] Yali AM, Lobel M (2002). Stress-resistance resources and coping in pregnancy. Anxiety Stress Coping.

[33] Cox J, Holden J, Sagovsky R (1987). Detection of postnatal depression. Development of the 10-item Edinburgh postnatal depression scale. Br J Psychiatry.

[34] Osborne RH, Batterham RW, Elsworth GR, Hawkins M, Buchbinder R (2013). The grounded psychometric development and initial validation of the health literacy questionnaire (HLQ). BMC Public Health.

[35] Rowlands IJ, Redshaw M (2012). Mode of birth and women’s psychological and physical wellbeing in the postnatal period. BMC Pregnancy Childbirth.

[36] Ng Fat L, Scholes S, Boniface S, Mindell J, Stewart-Brown SL (2017). Evaluating and establishing national norms for mental wellbeing using the short Warwick-Edinburgh Mental Well-being Scale (SWEMWBS): findings from the Health Survey for England. Qual Life Res.

[37] Herdman M, Gudex C, Lloyd A, Janssen M, Kind P, Parkin D (2011). Development and preliminary testing of the new five-level version of EQ-5D (EQ-5D-5L). Qual Life Res.

[38] Chesnut LW (2012). Centering pregnancy and adverse pregnancy outcomes: an evaluation of group prenatal Care in a Rural Western Kentucky Clinic: University of Alabama at Birmingham.

[39] Ickovics JR, Earnshaw V, Lewis JB, Kershaw TS (2016). Cluster Randomized Controlled Trial of Group Prenatal Care: Perinatal Outcomes Among Adolescents in New York City Health Centers. American Journal of Public Health.

[40] Heberlein EC, Picklesimer AH, Billings DL, Covington-Kolb S (2016). The comparative effects of group prenatal care on psychosocial outcomes. Arch Women’s Mental Health.

[41] National Collaborating Centre for Women’s and Children’s Health (UK) (2008). Antenatal Care: Routine Care for the Healthy Pregnant Woman London.

[42] Damschroder LJ, Aron DC, Keith RE, Kirsh SR, Alexander JA, Lowery JC (2009). Fostering implementation of health services research findings into practice: a consolidated framework for advancing implementation science. Implement Sci.

[43] Murray E, Treweek S, Pope C, MacFarlane A (2010). Normalisation process theory: a framework for developing, evaluating and implementing complex interventions. BMC Med.

[44] Sawtell M, Sweeney L, Wiggins M, Salisbury C, Eldridge S, Greenberg L (2018). Evaluation of community-level interventions to increase early initiation of antenatal care in pregnancy: protocol for the community REACH study, a cluster randomised controlled trial with integrated process and economic evaluations. Trials..

